# Supplementary material for: Fine-tuning foundational models to code diagnoses from veterinary health records
Source: PLOS Digit Health. 2026 Feb 20;5(2):e0001147. doi: 10.1371/journal.pdig.0001147 (PMC12923131; doi:10.1371/journal.pdig.0001147)
Supplement: Attachment — Submitted filename: Response to Reviewers.pdf [file pdig.0001147.s001.pdf]

Response to Reviewers

Adam Kiehl

Colorado State University

Adam.Kiehl@colostate.edu

June 6, 2025

Re: Manuscript ID PDIG-D-24-00525 – "Fine-tuning foundational models to code diagnoses from veterinary health records"

Dear PLOS Digital Health Editor,

We would like to thank you and the reviewers for your thoughtful and constructive feedback on our manuscript. We appreciate the time and effort that went into reviewing our work. Below, we provide a detailed, point-by-point response to each of the comments raised by the reviewers; track changes was used to identify changes to the manuscript.

Reviewer #1

Comment 1.1:

Although the authors refer to all tested architectures as "large language models," many (e.g., BERT base, GPT-2) fall into the smaller-to-mid-scale range by current standards. It would be more accurate to label them generally as pre-trained transformer models or small language models and reserve "LLM" for models of substantially larger parameter counts.

Response 1.1:

We have revised language throughout the manuscript to refer to all models as either simply "language models" or "foundational models."

Comment 1.2:

The paper focuses on the "Diagnosis" and "Assessment" EHR fields without deeply justifying why other sections (e.g., lab findings, treatment notes) are excluded. Additional sections might contain critical diagnostic indicators. A brief rationale for this decision, or experiments demonstrating that these two sections suffice for accurate classification, would strengthen the manuscript's methodological transparency.

Response 1.2:

Early on, we performed a sensitivity analysis to determine the optimal sections of clinical text to include in our training data by fine-tuning a model on different combinations of clinical text sections (results not shown). Of the fields available to us (diagnosis, assessment, presenting complaint, history, physical exam, and procedures and treatments), only *diagnosis* was capable of independently driving model performance. *Assessment* also demonstrated some performative capacity but not nearly as much as *diagnosis*. We include Table 4 to showcase that *diagnosis* is driving most of the performance. It was further found that concatenating many of the sections of clinical text together did not substantially improve model performance, likely due to the fact that the models used in this project are subject to constraints on the number of tokens that can be inputted. The *assessment* section was included in the training data to provide broader context for the visit and because there was still a low truncation rate with that text. Other sections were not included as they would often be truncated. This experiment is now briefly described in Materials and Methods/Data/Input Fields and Preprocessing. Future studies may explore models with larger context windows and this is now briefly mentioned in the Discussion/Future Work section.

Comment 1.3:

Some portion of the dataset (up to 13% of notes) was truncated to fit within the models' input limits, but the authors do not analyze how much this truncation might harm performance. Past research (e.g., Beltagy et al. 2020 on Longformer) demonstrates that transformer models with extended context windows often outperform those that must heavily truncate clinical notes. Examining alternative or more advanced approaches (e.g., chunking, hierarchical attention) would be beneficial. In the case of this paper, only 13% of notes were truncated, but in medicine, it is very common for notes to be longer than some of the limited context size.

Response 1.3:

We are cognizant of the potential consequences associated with truncation of input text and specifically chose to exclude many sections of clinical text to avoid excessive truncation (see Response 1.3). While 13% truncation is small, it is certainly worthy of consideration. The reason we are comfortable with that truncation rate is the fact that it is primarily the *diagnosis* section that drives model performance (see Response 1.3), and this section has a 0% truncation rate; only text from the *assessment* section is ever lost. We recognize that more advanced training techniques and model architectures may allow for more complete training data to be used, but we saw no strong indications that excluded sections of clinical text would greatly contribute to improved model performance. Our desire to engage with models with larger context windows in future is now expressed in the Discussion/Future Work section.

Comment 1.4:

The paper provides limited information on whether all layers were fine-tuned or only top layers. Techniques like layer-wise learning rate decay or partial freezing can impact performance and efficiency. More explicit detail on learning rates, epochs, and potential experiments comparing different fine-tuning strategies would aid reproducibility. This is particularly true for your "out-of-the-box" comparison, as you have to attach a classifier head, which means you are technically fine-tuning, just freezing the transformer layers.

Response 1.4:

We expanded our Materials and Methods/Models section to more completely describe our fine-tuning framework. We now specifically state that we fine-tuned all transformer blocks when feasible (some of our larger models could not be fine-tuned to the fullest extent); this choice was made through a trial-and-error process. In this section, we also include details concerning our appended layers, batch size, loss function, optimizer, learning rate, early stopping criteria, and maximum epochs. Additionally, we plan to make our codebase publicly available through GitHub, which will give developers access to our exact methodology.

Comment 1.5:

While the paper thoroughly reports predictive metrics, it lacks any discussion of interpretability methods. Explainability is crucial in medical or veterinary contexts to build trust and identify potential errors in model reasoning. Approaches such as attention visualization, Integrated Gradients, LIME, or SHAP (as in Mullenbach et al. 2018) could help users understand how the model arrived at a specific set of codes.

Response 1.5:

We recognize model explainability as a crucial component of both promoting trust in our model and enabling future model improvements. Thus far, we have implemented an integrated gradients method that attributes predictive importance to input tokens and have begun exploring these results internally. We intend to expand upon these efforts and perform a more in-depth analysis of the findings in a separate manuscript, as this work is beyond the scope of the current manuscript. Our progress and intentions to continue exploring model explainability are now presented in the Discussion/Future Work section.

Comment 1.6:

While the paper justifies the "out-of-the-box" comparison, freezing the weights and only implementing a classifier head, there has been significant research into how this creates unstable gradients and unpredictable (and challenging to reproduce) experiments. It is not surprising, and a very well-known scenario, that encoder models are used for different purposes, and each mode has different recommended pre-training and fine-tuning methods. Some of the failures of convergence are most likely related to these behaviors.

Response 1.6:
We appreciate the insight into the reasons for the poor model performance we observed in the out-of-the-box setting. We have now found a source that corroborates this narrative and cite it in the Results section. However, we also found sources that support the opposite finding. Therefore, we have decided to still include the out-of-the-box results table in our manuscript because we believe the experiment is justified.

Comment 1.7:
The paper does not present a detailed breakdown of which SNOMED-CT categories are more prone to errors. Analyses of precision/recall by diagnostic group (e.g., musculoskeletal, gastrointestinal, etc.) would reveal if the model struggles disproportionately with certain code sets. Understanding these weaknesses is often critical for targeted improvements and safer clinical adoption.

Response 1.7:
We also believe that a more comprehensive understanding of the strengths and weaknesses of our models is essential to proper and responsible application. We have begun exploration of model performance aggregated by disease category and body site, which we believe would be best presented as part of a more in-depth analysis of model performance in a separate manuscript. We have included our initial approach to breaking model results down by disease category as a table in the Methods/Performance Analysis section and briefly discuss these results and remaining work in the Discussion/Performance Analysis section.

Comment 1.8:
Differentiate between large-scale models (with billions of parameters) and smaller or mid-sized transformer-based architectures. Reserve the term “LLM” for the former, and use “pre-trained transformer” or “foundation model” more generally. Most of the work the paper could use would be “medium” at best. There is also a little discussion on MoE type models (albeit due to size) and “agentic” style models that should be discussed early on so users understand that you generally focus only on SLM/pre-trained models of the encoder only variety.

Response 1.8:
We have revised the manuscript to refer to all models as either simply “language models” or “foundational models” (see Response 1.1). We also now include a sentence in Materials and Methods/Models stating that ensemble, mixture of experts, and agentic models were not considered for this study. Several of the selected foundational models were of decoder-only variety, so we did not specify that only encoder-only models were considered.

Comment 1.9:

The fine-tuning methodology is unclear, with the only statement being as many as possible. There is a lot of work out there about how to fine-tune the non-vet models (i.e. VetBert and PetBert are not widely used), and the decision of how to or how not to fine-tune the model is critical for the experiment. Justification also needs to be given as to why, for the non-veterinary models, pretraining was not done. Some important questions needing answers:
- How many layers were the final models fine-tuned on?
- How many unknown/sub-word tokens per a model?
- Progressive unfreezing was not used because?
- Total number of epochs
- Gradient accumulation and gradient clipping parameters
- And a few others.

Response 1.9:

Please see Response 1.4 for a discussion of our documentation of fine-tuning methods. Some questions that are posed by this comment that were not present in Comment 1.4 cover topics such as additional pretraining, the rate of unknown token use, and progressive unfreezing. While additional pretraining was considered at the beginning stages of this project, it was decided that only fine-tuning should be performed to isolate the effect of fine-tuning on model performance. In the context of comparing model performance to VetTag, the pretraining that was performed by the Stanford University team has now been replaced by the pretraining performed to develop foundational language models. Secondly, the rate of unknown tokens used did not exceed 0.36% for any tokenizer; computing the rate of sub-word tokens used is not a simple prospect and therefore was not completed for this manuscript. We looked into implementing progressive unfreezing following your feedback and the implementation conflicted with both the distributed computing and scheduled optimization aspects of our training script. Due to these difficulties, we've decided not to implement progressive unfreezing for this study.

Comment 1.10:

Conduct a brief study of how truncating the text impacts coding accuracy. If truncation substantially degrades performance, adopting transformer architectures that can handle longer context windows—such as Longformer (Beltagy et al. 2020)—or chunking strategies might be beneficial.

Response 1.10:

Please see Response 1.3. We did not find any evidence that truncation meaningfully degraded model performance and so additional steps were not taken to utilize models with larger context windows.

Comment 1.11:

The authors have already started discussing the relationship of F1 score to prediction terms. Still, word cloud analysis, syllable/complexity analysis, and other standard explainability tools (LIME/SHAP) should also be considered to understand why the algorithms output their predictions. Particularly if there are patterns of input that seem to result in different outputs.

Response 1.11:

Please see Response 1.5. We have begun to implement methods for model explainability (integrated gradients) but would like to develop a more comprehensive narrative surrounding the idea of explainability before publishing much of our work. We do now acknowledge that progress has been made in this area in the Discussion/Future Work section of our manuscript, although this work is largely beyond the scope of this manuscript.

Comment 1.12:

Present an analysis that groups SNOMED-CT codes into broader categories and reports classification metrics for each. It is not necessary to do an "in-depth" analysis on this part, but if there are clusters, particularly around veterinary only or human diseases that behave differently in animals, this would be important. There is a lot of work around noun-confusion in transformers and knowing this is a critical component to understanding performance.

Response 1.12:

Please see Response 1.7. We have begun to implement methods for model performance categorization but would like to develop a more comprehensive narrative surrounding the idea of performance analysis before publishing our work. We do now acknowledge that progress has been made in this area in the Results/Performance Analysis and Discussion/Performance Analysis sections of our manuscript.

Comment 1.13:

Using the pooler layer, may result in poor performance independent of mode. Pooler layers have weights and biases that are directly related to how the "Vanilla" model was pre-trained and it's downstream tasks. I would recommend not using it for 2 reasons, 1) not all models have it so additional future comparison is challenging and 2) the biases are a known issue and impact downstream task adoption. Consider using a FC with a normalization layer to the last hidden state of the transformer model for a more fair comparison.

Response 1.13:

We thank the reviewer for pointing out this flaw in our training methodology. Previously, some models had pre-trained poolers and some had randomly initialized poolers. To

address this inconsistency between models, we dropped all pre-trained poolers and implemented custom poolers (a fully-connected linear layer followed by a Tanh activation function) for all models, per the reviewer's recommendation. This process is described in Materials and Methods/Models. All models have been re-trained, and the results in Table 2 are up to date and improved slightly.

Comment 1.14:

There has been a lot of work in the past year or so on architectures that change how attention is utilized and have significantly improved performance beyond vanilla BERT. He, et al 2023 for DeBERTaV3 and Warner, et al 2024 for ModernBERT show many of the flaws that current BERT architectures has and particularly the problem you are experiencing in the analysis are discussed in both papers. I strongly encourage the authors to reconsider their model choices as while veterinary medicine is "new," there has been much work in this area already, and we can leverage that to skip the issues. I would reduce the number of models tested and improve the depth of analysis as the broad stroke process for known poor-performance (and vanilla out of the box) reduces the potential paper's impact.

Response 1.14:

We recognize that the scope of foundational models considered in this project is somewhat limited, despite our attempts to capture a wide variety of model sizes and pre-training data. While we did not remove the older BERT models from our paper in the interest of total transparency, we did add both DeBERTa V3 and ModernBERT. They are now both fine-tuned under the same framework as all other models and classification results are reported in Table 2. These additional models are now referenced in Introduction/Related Work/Non-Clinical LMs, Materials and Methods/Models, and Discussion.

Comment 1.14:

Additional References to Review and Incorporate:

- Explainability in Clinical NLP Mullenbach et al. 2018
- Long Document Transformers for Medical Text Beltagy et al. 2020
- Smarter, Better, Faster, Longer: A Modern Bidirectional Encoder for Fast, Memory Efficient, and Long Context Finetuning and Inference Warner et al. 2024.
- DeBERTaV3: Improving DeBERTa using ELECTRA-Style Pre-Training with Gradient-Disentangled Embedding Sharing He et al. 2023
- Transformer models in biomedicine Madan et al. 2024
- Task-Specific Transformer-Based Language Models in Health Care: Scoping Review Cho, et al. 2024
- Transformer Models in Healthcare: A Survey and Thematic Analysis of Potentials, Shortcomings and Risks Denecke et al 2024
- A comparative study of pretrained language models for long clinical text Li et al. 2022
- TransformEHR: transformer-based encoder-decoder generative model to enhance prediction of disease outcomes using electronic health records Yang et al. 2023
- A Pretrainer's Guide to Training Data: Measuring the Effects of Data Age, Domain Coverage, Quality, & Toxicity Longpre et al. 2024

Response 1.15:

We thank the reviewer for the additional sources provided for our consideration. Many have now been appropriately cited in our manuscript.

Comment 1.16:

Minor corrections:

1. Some of the sentences are particularly long with the usage of i.e. Consider removing the i.e. on repeated usage or in many cases since the paper is trying to generalize, use e.g. as the preferred term.
2. Figure 2 text is very long, describing a "result" of success on that note. While interesting, the point of Figure 2 is to show the "data." It may be beneficial to show examples of cases that all algorithms could get when they were vanilla vs fine-tuned, but this would be nice to have.
3. On Page 18, The graph has ln() number of records, is there supposed to be a unit, like x1k records? If not, remove it.
4. On page 19, the Figure 4 definition is super long. Some of that text should be in the discussion of the figure and not in the figure text. Consider shortening the Figure 4 description and moving the valuable discussion to the discussion text directly.

Response 1.16:

We have considered and implemented many of these corrections to our manuscript. The captions on Figures 2 and 4 have been truncated and some inappropriate uses of i.e. have been removed. The axis label on Figure 3 indicates that the unit of the x-axis is the natural log of the number of records that each diagnosis appears in. We have modified the axis label slightly to accurately reflect the intended units.

Reviewer #2

Comment 2.1:

It's understandable that the data used to train the model cannot be made available, but the model itself or any ability to evaluate the model should be made available, as was the case for both VetBERT and PetBERT. Otherwise, the study is only pointing to a model that is theoretically better than others without any way to evaluate. At a minimum there should be a section explaining why the model is not being made available.

Response 2.1:

We respect the open science process and wish to do everything we reasonably can to promote research transparency and reproducibility. We wanted to do our due diligence with respect to data security before making any of our materials public. We've been unable to reconstruct original fine-tuning data using our trained models. Therefore, our best fine-tuned model will be made available through PhysioNet with appropriate data/material use agreements and our training code will be made available through GitHub. Our model is currently under review by the PhysioNet team, and the correct project link will be added to the manuscript before publication. Our fine-tuning data will remain private due to privacy concerns and hospital policy. This position is stated in the Data Availability, Model Availability, and Code Availability sections.
